# Rates of assaults among New York City Transit workers before and during the COVID-19 pandemic, 2018 – 2023

**DOI:** 10.21203/rs.3.rs-9867527/v1

**Published:** 2026-06-05

**Authors:** Devan Hawkins, Michael Cziner, David Vlahov, Jonathan Rosen, Robyn R. M. Gershon

**Affiliations:** Massachusetts College of Pharmacy and Health Sciences New England School of Acupuncture: MCPHS University New England School of Acupuncture; New York University School of Global Public Health; Yale University School of Nursing; AJ Rosen & Associates LLC, Schenectady, NY; New York University School of Global Public Health

**Keywords:** transit workers, physical assault, workplace violence, COVID-19, temporal trend

## Abstract

Violence associated with the COVID-19 pandemic in non-health care essential workers remains underexamined. To address this gap, workplace violence trends in New York City (NYC) transit workers were investigated before, during and after the acute phase of the COVID-19 pandemic. Data on workers’ physical assault injuries were extracted from occupational injury and illness recordkeeping logs. Weekly physical assault rates for NYC subway and bus workers, separately and jointly, were calculated per 10 million transit riders, pre, peri, and post- pandemic (2018–2023), with trends analyzed using joinpoint regression and interrupted-time series analyses. Calculated trends of physical assaults on NYC transit workers per 10 million riders per week indicated a rate of 0.91 between January 2018 and the end of February, 2020. Starting with the onset of COVID-19 in NYC at the beginning of March 2020, amid record-low ridership, the rate rapidly increased to 11.91. By September, 2020, rates decreased to 4.77. These temporal patterns were similar for both bus and subway workers, however, rates were higher among bus workers. The observed spike in workplace assaults during the early acute phase of COVID-19 in 2020 was significantly beyond earlier generally rising trends in NYC transit assaults. Renewed attention to the risk and risk factors for violence targeting transit and other essential workers in general, and most especially during periods of large-scale societal disruptions, is warranted.

## 1. Introduction

In New York City (NYC), during the early phase of the COVID-19 pandemic, leadership at the city and state level vowed to keep the Metropolitan Transportation Authority (MTA) transit system operational to enable the essential workforce employed in critical infrastructure industries to continue commuting to their jobs. On March 22, 2020, at 8pm, the executive order, “The NYC Pause,” issued by then Governor, Andrew Cuomo, went into effect to try and stop the exponential spread of COVID-19. The order resulted in the closing of non-essential workplaces, schools, religious in-person services, social gatherings, and halted most forms of public contact, essentially ushering most New Yorkers into quarantine or home isolation.^1^ The effect on NYC public transit ridership was immediate. The system, one of the world’s largest, with over 1 billion annual ridership, decreased by nearly 75% overnight.^2^ Transit workers, however, were required to keep the system operating, as per United States (US) critical infrastructure rules (CISA) rules.^3^ Transit workers are considered essential because they are employed by one of the 16 critical infrastructure sectors whose “assets, systems, and networks, whether physical or virtual, are considered so vital to the United States that their incapacitation or destruction would have a debilitating effect on security, national economic security, national public health or safety, or any combination thereof.”^4^

Early in the COVID-19 pandemic data from an early study conducted in collaboration with Transport Workers Union, local 100, found that by August, 2020, more than one quarter of the roughly 70,000 MTA transit workers reported an episode of COVID-19 infection, 10,000 had been placed on home quarantine, hundreds were hospitalized with infection and more than 125 had died from the infection.^5^ One study showed that NYC transit workers were not only at high risk for acquiring COVID-19 infection, but that the peak of transit worker COVID-19 cases preceded the general population by just over a week.^6^

With growing widespread awareness about occupational exposure to COVID-19 among public-facing essential workers during the early phases of COVID-19, additional reports emerged on rising incidence of workplace violence.^7–9^ Increased violence during previous outbreaks, epidemics, and pandemics, collectively referred to as “bioevents,” has been documented^10,11^ Bioevents generally lead to societal disruptions and perturbations, such as loss of income, and can instill fear and anxiety, which has been linked to increased societal violence.^12^

While considerable attention has been shared regarding physical assaults on health care workers^7,8^, reports are sparse with respect to other essential workers, such as retail workers.^9,11^ Information on violence against transit workers was mainly reported in news reports during this period.^13^ The purpose of this study was to examine and describe the trends in physical assaults directed at NYC transit workers before, during and since the peak of the COVID-19 outbreak in 2020.

## 2. Methods

Under the authority of the New York (NY) State Public Employee Safety & Health (PESH) Act, and the NYS Workplace Prevention Act, the NY Metropolitan Transportation Authority (MTA) collects workplace violence data for NYC transit workers and records it on an occupational injury and illness log form (SH-900), which is equivalent to the Occupational Health and Safety Administration (OSHA 300).^14,15^ Deidentified data from these logs spanning from 2018 to 2023 were analyzed. These logs contained information on the incident, date, location, occupation of affected transit worker, lost work time, and a brief description of the incident. For this study we focused on assaults as defined by the Bureau of Labor Statistics as: “an intentional act of violence, threat of attack, or intimidation by another person” that were serious enough to require more than simple first-aid for bus and subway workers.”^16,17^

The first level of analysis was to calculate the annualized rate for number of assaults per 100,000 workers for all transit workers combined, and then separately, for subway and bus workers. The worker population data for 2022 was used throughout the surveillance period because accurate headcount data for other years were unavailable, and the union research staff who provided these data reported that there was no meaningful change during the study period, except for the immediate effect of home quarantine or isolation of workers during the first three months of the pandemic (March, 2020-June, 2020), when ridership also was markedly reduced.

Because denominator data for the worker population was based on a single year, we turned to examining daily subway and bus ridership numbers accessed through publicly available data from the MTA. Another rationale for turning to ridership data was that it reflected a key element in evaluating assaults: exposure to the potential source of violence; i.e., the riders.

Daily ridership estimates were available every day from March 1, 2020, to December 31, 2023. For data before March 1, 2020, we used values published by the MTA for the average weekday, Saturday, and Sunday ridership for 2018 to 2019.^18^ For data from 2018 to 2023, we converted daily data to weekly data to estimate the weekly number of assaults per 10 million riders each week from 2018 to 2023.

We used two approaches to examine changes in weekly assault rates: Joinpoint regression and interrupted time series analyses. Joinpoint regression enables modeling of trends in time-series data using multiple straight-line segments connected at “joinpoints,” which can identify statistically significant changes in the trend’s direction or rate.^19^ Joinpoint regression was used because we were particularly interested in identifying points when changes occurred due to the dramatic drop in ridership during the early dates of the COVID-19 pandemic, starting with reports in early March, 2020^20^ of the first confirmed case of COVID-19 in a subway rider,^21^ followed soon after by a reduction in ridership which lasted for several months. For the analysis combining the bus and subway workers, we set the parameters to identify a maximum of 4 joinpoints. The joinpoint analysis was performed for the bus and subway workers combined and then separately. The results from this analysis informed the dates that were examined as part of the interrupted time series analysis, which was conducted to identify changes in both the absolute rate of assaults and changes in trends.

Based on the joinpoint analysis for bus and subway workers combined, it was found that rates started to increase from week 10 of 2020 to week 18 of 2020, before decreasing until Week 38 of 2020. Therefore, in the interrupted time series analysis,^22^ we examined two separate interruptions: week 10 of 2020 and week 38 of 2020. Due to the significant changes in ridership in March 2020 (week 10), we investigated the extent to which changes in the number of assaults, independent of ridership, may have influenced our findings. Using the average weekly number of assaults between 2018 and 2019 for bus and subway workers separately and combined as a baseline, we then examined the number of weeks from 2020 to 2023 that exceeded this average by at least two standard deviations.

## 3. Results

### Descriptive Analysis

Between 2018 and 2023, there were a total of 2,454 reported assaults among NYC bus and subway workers. Of these, 1,350 assaults occurred among bus workers and 1,104 among subway workers. Based on headcount data from 2018 (10,647 bus workers and 24,489 subway workers), there were 21.1 assaults per 1,000 bus workers per year (95% confidence interval (CI) = 20.0, 22.3) and 7.5 assaults per 1,000 subway workers per year (95% CI = 7.1, 8.0) from 2018 to 2023. Using total ridership as the denominator, there were 0.78 assaults per 10 million bus riders per year (95% CI = 0.74, 0.82) and 0.26 assaults per 10 million subway riders per year (95% CI = 0.25, 0.28) over the same calendar period.

### Joinpoint Analysis

**Figure 1.**
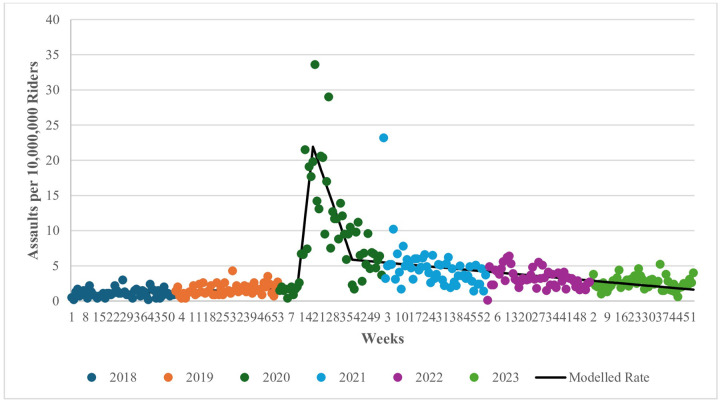
Assaults per 10 million riders among New York City bus and subway workers by week, 2018 to 2023, showing four distinct trends identified by Joinpoint analysis

Table 1 presents the temporal cutoffs from the joinpoint analysis of assaults among the combined NYC Transit bus and subway workers from 2018 to 2023. Depicted in Fig. 1, four distinct trends were observed. From the start of 2018 to Week 10 of 2020, a flat trend was observed during the onset of the COVID-19 pandemic. Between Week 10 and Week 18 in 2020, there was a very rapid increase, followed by a sharp decline from Week 18 to Week 38 of 2020. From Week 38 of 2020 to the end of 2023, there was a gradual, though statistically significant, decreasing trend.

We proceeded to examine the joinpoint separately for each of the two worker categories (Table 2). For bus workers, five distinct trends were identified, including a gradual, non-significant increasing trend that concluded in week 13 of 2020 (Supplemental Fig. 1). There was a rapid increase in the rate of assaults from weeks 13 to 18 of 2020. Unlike the pattern observed for the combined bus and subway workers, two declining trends were noted: one with a significant declining slope from week 18 to week 33 of 2020 and another with a much steeper but not statistically significant decline from week 33 to week 36 of 2020. Consistent with the overall data for the latter part of 2020 through the end of 2023, an essentially flat slope was observed. For subway workers (Supplemental Fig. 2), between week 11 to week 14 in 2020, a rapid, although statistically non-significant increase was seen followed by a statistically significant decline between weeks 14 to 43 in 2020. Afterwards, a gradual, statistically significant decline occurred between week 43 of 2020 and the end of 2023.

### Interrupted Time Series Analysis

**Figure 2.**
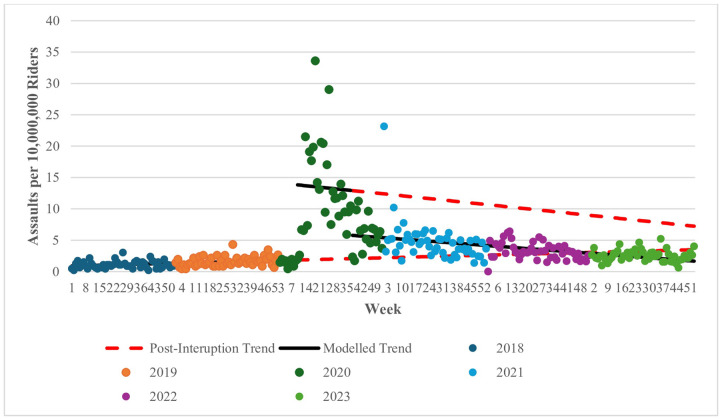
Interrupted time series analysis of assaults per 10 million riders among combined New York City Bus and Subway Workers by week, 2018 to 2023

Table 3 presents the coefficients for intercepts and trends from the interrupted time series analysis of assaults among NYC bus and subway workers from 2018 to 2023. Figure 2 and Supplemental Figs. 3 and 4 depict these trends where the solid lines reflect models based on the data within the intervals identified earlier through the joinpoint analyses. The dashed lines represent the trends modelled to depict trajectory of data if the pre-interruption interval continued.

For bus and subway workers combined (Fig. 2), in week 1 of 2018, the predicted rate of assaults was 0.91 per 10 million riders (95% CI = 0.69, 1.12). Between week 1 of 2018 and week 10 of 2020, this rate experienced a gradual, statistically significant increase. In week 10 of 2020, at the onset of the COVID-19 pandemic, there was a substantial increase in the rate:11.98 (95% CI = 5.69, 18.27). Following this increase, a non-significant gradual decline occurred from week 10 to week 38 of 2020. In week 38, another statistically significant change in the intercept took place, shown by a decrease of −7.14 (95% CI= −11.31, −2.97). From week 38 to the end of 2023, there was a gradual, statistically significant decline in the rate.

For bus workers (Supplemental Fig. 3), the modeled intercept at the beginning of 2018 was higher than that for bus and subway workers combined (1.79; 95% CI = 1.31, 2.27). Supplemental Fig. 3 shows the rates depicted on a log scale due to the extreme increase in rates during the pandemic period. The trend for bus workers between the beginning of 2018 and week 10 of 2020 mirrored the gradually increasing trend observed for the combined bus and subway workers. The increase in the modeled intercept following the interruption in week 10 of 2020 was more than 17 times greater than that observed for bus and subway workers combined. After this interruption, a non-significant increasing trend was noted through week 38 of 2020. By week 38, another substantial and significant interruption occurred, with a decrease in the modeled intercept of −213.02 (95% CI = −336.88, −89.16). Similar to the findings for combined bus and subway workers, from week 38 of 2020 to the end of 2023, there was a statistically significant, gradually declining trend.

For subway workers (Supplemental Fig. 4), the modeled intercept in week 1 of 2018 was lower than that of bus workers (0.38, 95% CI = 0.25, 0.51) with a flat trend until week 10 of 2020. The intercept change in week 10 of 2020 for subway workers was substantial, though much less than the change observed for bus workers. Following this interruption, there was a non-significant decline until week 38 of 2020. At week 38, the intercept declined significantly (−4.18, 95% CI= −6.66, −1.70). As observed in the combined findings and the results for bus workers from week 38 of 2020 to the end of 2023, no significant decline was observed.

Another way to depict the temporal patterns of assaults was to display the number of weeks during a year in which assaults were 2 standard deviations or more above the average for the period from 2018 to 2019 (Table 4). This approach, which examines only counts of assaults, was used because the rapid decline in ridership during COVID-19 could have been the main contributor to the increase in rates. For bus and subway workers combined, only weeks 1 and 2 had the number of assaults exceed this threshold in 2018 and 2019, respectively. However, in 2020, 6 weeks (11.3%) surpassed this threshold, all occurring after the onset of the acute phase of the pandemic in March (13.6% of the acute pandemic weeks). In 2021 and 2022, the number of assaults exceeded this threshold, with 9 (17.0%) in 2021 and 7 (13.2%) in 2022. The number declined to 3 weeks that exceeded this threshold in 2023 (5.7%), which was still greater than in either of the pre-2020 years. The pattern was even more pronounced for bus workers with no weeks exceeding the threshold in 2018 and 3 weeks exceeding it in 2019. Over one-fifth of the weeks (12, 22.6%) surpassed the threshold in 2020, all occurring during the acute phase of the pandemic (27.3% of the acute pandemic weeks). Although the number of weeks declined, it remained elevated compared to the pre-2020 period in 2021 (10, 18.9%), 2022 (9, 17.0%), and 2023 (5, 9.4%). For subway workers, only 1 week exceeded the threshold in 2018 (1.9%), and 3 weeks exceeded it in 2019 (5.7%). In 2020, 7 weeks exceeded the threshold, all occurring during the acute phase of the pandemic (15.9% of the acute pandemic weeks). The number of weeks exceeding the threshold declined in 2021 (4, 7.5%), then increased in 2022 (8, 15.1%) before declining again in 2023 (4, 7.7%).

## 4. Discussion

The major finding of this study was the spike in the rate of assaults among NYC transit workers during the early phase of the COVID-19 epidemic in New York City. While the rates subsided following the acute phase, physical assaults among NYC transit workers remained elevated in December 2023 compared to January 2018. These increased rates of assault are especially notable given the fact that ridership on New York City transit systems declined substantially during the COVID-19 pandemic; in April 2020, the NYC transit ridership was only 8.3% of the April, 2019 levels.^23^ The lack of ridership may have increased the risk of danger to transit workers during a time of general unease throughout New York City.

Another finding was the similar trend but higher rate of assaults among bus than subway workers. This may have been due to bus workers’ closer proximity to passengers. In September 2023, the MTA sought to address assaults on bus operators by introducing a new prototype cockpit to enhance the physical barrier between passengers and the operator.^24^ While the degree to which these steps affected rates remains an open question, results from Table 3 suggest a significant drop in assaults on bus workers in the final part of the study period. Steps were also taken to reduce violence against subway workers; the MTA worked with city officials to increase police deployment in the subway system.

Important differences between the rates presented here and other official sources requires examination. For example, our findings differ from those of the US National Transit Database (NTD), which showed a decrease in 2020 and an increase afterwards that exceeded the pre-COVID-19 period.^25^ The difference may be due to several factors, including the fact that the NTD represents national data rather than NYC data. Another reason for a difference between the OSHA SH-900 data and the NTD data is that the NTD data on assaults reflects only deaths and cases requiring transport to hospitals. In contrast, the SH-900 data represents a wider range of physical assault events, including those that did not require transport or result in a fatality. Finally, the reporting forms and mechanism for NTD forms S&S-40 and S&S-50, differ from the SH-900 logs.^25^ Our findings also differ from the New York Police Department (NYPD) data, which noted an increase every year from 2019 (94 assaults) to 2023 (168 assaults).^26^ The discrepancy between the police data and the SH-900 data might have resulted because assaults reported via SH-900 were not necessarily reported to the police. At face value, these reporting discrepancies could indicate that the severity of non-reported physical assaults reported in our study was less extreme than those that were reported to the NYPD. Additionally, the US Public Employee Safety and Health criteria to report under the public employer workplace violence prevention standard, New York State Labor Law 27b,^27^ is different than the NYS Penal Law.^26^ Studies also suggest that many workers are reluctant to report assaults to the police due to factors such as minor injury, lack of support by employers, and belief that reporting will not lead to positive change. ^28^

A potential limitation in our analysis is the lack of precise headcount numbers. Although we were informed that the headcount remained stable across the study period, the lack of more specific information can produce only approximate rates. Additionally, headcounts as a denominator may not account for all of the time possibly worked, such as overtime or lost days. Furthermore, the logs would not contain assaults in the event that the injury was not reported. Underreporting is a known major issue in occupational health studies.^28^

Changes in reporting might also have occurred as a result of new legislation promulgated in 2022, when the NYS Governor extended assault in the 2nd degree (a felony charge) against individuals who assaulted station customer assistants, ticket or revenue collectors, maintenance workers, repairers, cleaners, and their supervisors.^29^ Previously, only bus operators and subway conductors and operators were protected by the law.^29^

Despite differences between the reporting agencies, the overall findings suggest the need for more rigorous assessment of risk factors for violence directed at public transit workers. Despite the 2006 passage in New York State Workplace Violence law (Labor Law 27-b),^27^ which stipulated important violence prevention interventions: record keeping, reporting, risk assessments, training requirements for public and public authority workers, and an annual review of the workplace violence prevention plan by management and union representatives, workplace violence overall has continued to be a growing problem.^30,31^

More recent legislation aims to reduce the risk in transit. On September 25, 2024, the FTA issued General Directive 24 – 1: Required Actions Regarding Assaults on Transit Workers.^32^ This General Directive requires transit agencies subject to FTA’s Public Transportation Agency Safety Plans to conduct a safety risk assessment, identify safety risk mitigations or strategies and provide information to FTA on how they are assessing, mitigating and monitoring the safety risk associated with assaults on transit workers.^32^ This type of detailed data collection regarding occupational violent injuries – such as the perpetrator, the mode of the assault, and precipitating factors – may help to better inform interventions to improve worker safety and can be key to protecting the wellbeing of transit workers as well as the riding public. Further, any time there is a new risk factor identified, the FTA regulations require an assessment and establishment of feasible protective measures. Future studies should explore the impact of these relatively new initiatives, as well as the potential role of engineering controls to physically separate bus drivers and subway workers from potential perpetrators of violence, as well as the impact of increased presence of police in reducing assaults. A relatively recent review of measures to prevent and address violence directed against transit workers supports the use of multiple strategies to reduce risk of violence, including video surveillance, de-escalation techniques, as well as physical barriers.^33^ Additional research is needed on these strategies, specifically for the transit environment during public health crises, for which data are scant.^34^

The data presented here indicate that the COVID-19 pandemic’s toll extended beyond viral-associated morbidity and mortality. Our research adds to a body of literature that suggests that a bioevent (i.e., the COVID-19 pandemic) can foster an atmosphere leading to increased violence. Cohn and Kutalek^35^ and Whiteman and colleagues^10^ provide a good discussion of the risk factors for increased violence during bioevents, including increased disruption of normal social contacts, social isolation of potential victims, disrupted social networks, lack of needed psychological and psychiatric care for unwell perpetrators, and poor implementation of control measures. It is critical that employers and government officials learn from the COVID-19 experience to better protect the well-being of front-line essential workers, such as healthcare, transit, retail workers, and others who were subjected to increased acts of violence, while putting their lives at risk by reporting for duty.

## Conclusion

Transit workers experienced a spike in workplace assaults during COVID-19, significantly beyond the earlier rising trend in assaults. While this pattern is consistent with similar increases in violence directed at other essential worker populations during bioevents, renewed attention to improving situational awareness and promoting incident prevention through appropriate interventions for this population of transit workers is indicated. Referred to as the “pandemic within the pandemic,”^10^ the issue of concomitant violence during bioevents needs to be urgently addressed in disaster risk management planning, before the next bioevent.

## Supplementary Material

Supplementary Files

This is a list of supplementary files associated with this preprint. Click to download.

- SupplementalFigure1Transitnew.jpg
- SupplementalFigure2Transitnew.jpg
- SupplementalFigure3Transitnew.jpg
- SupplementalFigure4Transitnew.jpg

## Figures and Tables

**Table 1:** Results of joinpoint analysis of assaults combined New York City bus and subway workers, 2018–2023

Segment	Period	Slope (95% CI)	P-value	Slope Change (95% CI)	P-value
1	Week 1 2018-Week 10 2020	0.01 (0.00, 0.02)	0.193		
2	Week 10 2020-Week 18 2020	2.51 (1.54, 3.48)	< 0.001	2.50 (1.54, 3.47)	< 0.001
3	Week 18 2020-Week 38 2020	−0.80 (−0.99, −0.61)	< 0.001	−3.31 (−4.30, −2.33)	< 0.001
4	Week 38 2020-Week 53 2023	−0.02 (−0.03, −0.02)	< 0.001	0.78 (0.59, 0.97)	< 0.001

**Table 2:** Results of joinpoint analysis on assaults separately for New York City bus and subway workers, 2018–2023

	Segment	Period	Slope (95% CI)	P-value	Slope Change (95% CI)	P-value
**Bus Workers**	1	Week 1 2018-Week 13 2020	0.01 (−0.19, 0.20)	0.941		
	2	Week 13 2020-Week 18 2020	84.75 (40.56, 128.94)	< 0.001	84.74 (40.55, 128.93)	< 0.001
	3	Week 18 2020-Week 33 2020	−15.45 (−20.73, −10.16)	< 0.001	−100.19 (−144.7, −55.69)	< 0.001
	4	Week 33 2020-Week 36 2020	−61.70 (−209.15, 85.74)	0.413	−46.26 (−193.79, 101.28)	0.539
	5	Week 36 2020-Week 53 2023	−0.04 (−0.14, 0.07)	0.518	61.67 (−85.77, 209.11)	0.413
**Subway Workers**	1	Week 1 2018-Week 11 2020	0.00 (0.00, 0.01)	0.251		
	2	Week 11 2020-Week 14 2020	3.17 (−2.61, 8.95)	0.284	3.16 (−2.62, 8.95)	0.284
	3	Week 14 2020-Week 43 2020	−0.28 (−0.35, −0.21)	< 0.001	−3.45 (−9.23, 2.33)	0.243
	4	Week 43 2020-Week 53 2023	−0.01 (−0.01, 0.00)	0.001	0.28 (0.21, 0.34)	< 0.001

**Table 3:** Results from Interrupted time series analysis of assaults among New York City Bus and Subway workers, 2018–2023

	Bus and Subway Workers		Bus Workers		Subway Workers	
	Coefficient (95% CI)	P-value	Coefficient (95% CI)	P-value	Coefficient (95% CI)	P-value
**Intercept** (Week 1 2018)	**0.91** (0.69, 1.12)	< 0.001	**1.79** (1.31, 2.27)	< 0.001	**0.38** (0.25, 0.51)	< 0.001
**Trend:** (Week 1 2018–Week 10 2020)	0.01 (0.00, 0.01)	< 0.001	0.01 (0.01, 0.02)	< 0.001	0.00 (0.00, 0.01)	< 0.001
**Intercept change** (Week 10 2020)	**11.98** (5.69, 18.27)	< 0.001	**207.19** (60.34, 354.04)	0.006	**6.30** (2.48, 10.11)	0.001
**Trend change** (Week 10 2020)	−0.04 (−0.37, 0.29)	0.808	0.42 (−7.88, 8.72)	0.921	−0.03 (−0.23, 0.16)	0.743
**Trend** (Week 10 2020–Week 38 2020)	−0.03 (−0.36, 0.30)	0.846	0.43 (−7.87, 8.73)	0.919	−0.03 (−0.22, 0.17)	0.777
**Intercept change** (Week 38 2020)	**−7.14** (−11.31, −2.97)	0.001	**−213.02** (−336.88, −89.16)	0.001	**−4.18** (−6.66, −1.70)	0.001
**Trend change** (Week 38 2020)	0.01 (−0.32, 0.34)	0.957	−0.47 (−8.77, 7.84)	0.913	0.02 (−0.17, 0.21)	0.834
Week 38 2020-Week 53 2023	−0.02 (−0.03, −0.02)	< 0.001	−0.03 (−0.05, −0.02)	< 0.001	−0.01 (−0.01, 0.00)	< 0.001

**Table 4:** Number of weeks where the number of assaults was 2 standard deviations or more above the average for the period from 2018 to 2019 among New York City bus and subway workers, 2018 to 2023

	Year	Number of weeks exceeding 2 SDs	Total Weeks	Percent of weeks
Bus and Subway Workers	**2018**	1	53	1.9
	**2019**	2	53	3.8
	**2020**	6	53	11.3
	**2020 (March to January)**	6	44	13.6
	**2021**	9	53	17.0
	**2022**	7	53	13.2
	**2023**	3	53	5.7
	**Total**	28	318	8.8
	**2018**	0	53	0
	**2019**	3	53	5.7
	**2020**	12	53	22.6
Bus Workers	**2020 (March to January)**	12	44	27.3
	**2021**	10	53	18.9
	**2022**	9	53	17.0
	**2023**	5	53	9.4
	**Total**	39	318	12.3
	**2018**	1	53	1.9
	**2019**	3	53	5.7
	**2020**	7	53	13.2
Subway Workers	**2020 (March to January)**	7	44	15.9
	**2021**	4	53	7.5
	**2022**	8	53	15.1
	**2023**	4	52	7.7
	**Total**	27	318	8.5
